# Risk of Injury and Mortality among Driver Victims Involved in Single-Vehicle Crashes in Taiwan: Comparisons between Vehicle Types

**DOI:** 10.3390/ijerph17134687

**Published:** 2020-06-29

**Authors:** Ya-Hui Chang, Chung-Yi Li, Tsung-Hsueh Lu, Kurnia Dwi Artanti, Wen-Hsuan Hou

**Affiliations:** 1 Department of Public Health, College of Medicine, National Cheng Kung University, Tainan 701, Taiwan; yhccat@gmail.com (Y.-H.C.); cyli99@mail.ncku.edu.tw (C.-Y.L.); robertlu@mail.ncku.edu.tw (T.-H.L.); 2 Department of Public Health, College of Public Health, China Medical University, Taichung 404, Taiwan; 3 Department of Healthcare Administration, College of Medical and Health Science, Asia University, Taichung 413, Taiwan; 4 Department of Epidemiology, Faculty of Public Health, Universitas Airlangga, Surabaya 60115, Indonesia; kurnia-d-a@fkm.unair.ac.id; 5 Department of Physical Medicine and Rehabilitation, Taipei Medical University Hospital, Taipei 110, Taiwan; 6 Master Program in Long-Term Care & School of Gerontology Health Management, College of Nursing, Taipei Medical University, Taipei 110, Taiwan; 7 Graduate Institute of Clinical Medicine, College of Medicine, Taipei Medical University, Taipei 110, Taiwan; 8 Center of Evidence-Based Medicine, Taipei Medical University Hospital, Taipei 110, Taiwan

**Keywords:** injury, mortality, gender, traffic accident

## Abstract

Vehicle-type specific injury severity has rarely been investigated mainly because of a lack of such information in hospital-based studies that normally exclude those who are severely injured and die on the scene. No study has been conducted either on driver characteristics in single vehicle crashes in Taiwan according to vehicle type. This was the first population-based study aiming to describe demographic characteristics in association with vehicle-specific rates of injury and fatality among driver victims involved in single-vehicle crashes in Taiwan. We presented sex and age-specific number and proportion of driver victims according to vehicle type. We calculated sex and age-specific rates of injury and fatality. Injury and fatality rates were also graphically presented. Bicycle and motorcycle rider victims generally had higher injury rates but lower fatality rates. However, older (45+) bicycle rider victims had greater fatality risk. By contrast, truck and car driver victims were generally associated with lower injury rates but with higher fatality rates. Elderly (65+ years) truck driver victims suffered from higher rates of injury and fatality. Male victims were found to have a higher fatality rate than female victims regardless of vehicle type. The vehicle-type-specific analyses of injury and fatality are considered useful in identifying single-vehicle crash victims at greater risks of injury and fatality.

## 1. Introduction

The World Health Organization claimed a rise in road traffic deaths of more than 1.35 million in 2016. Road traffic is the leading cause of death among younger people aged between 5 and 29 years. Despite these massive and largely preventable losses in human lives and consequent toll, actions to combat this global challenge have been insufficient [1]. Taiwan road traffic victims dying within 30 days was 11.44 per 100,000 persons, and in Korea and Japan were 8.1 and 3.7 per 100,000 in 2017, respectively [2,3]. Taiwan road traffic mortality figures are higher than in other Asian countries. In fact, the traffic accident fatality rate fell slightly per year in Taiwan, but the injury rate rose rapidly. [2]. Therefore, to develop strategies to solve this issue is important in Taiwan.

Analyses of injury and death among driver victims involved in single-vehicle crashes for specific vehicle types have important implications in terms of managing traffic safety measures and reducing the occurrence of critical traffic accidents. Previous studies showed that the factors contributing to injury severity may vary with vehicle type [4]. Yau also indicated that driver age has no significant effect on injury severity in an accident regardless of vehicle type (i.e., private vehicle, goods vehicle, motorcycle). Driver gender was also found to be a significant factor in injury severity only for private vehicles. These findings emphasize the need for the analysis of vehicle-type-specific injury severity, which was previously rarely conducted primarily due to limited information in hospital-based studies [5,6]. Martensen and Dupont (2013) collected databases from six European countries and showed heavy good vehicles and motorcycles were less likely to be involved in single vehicle fatal road crashes than cars [7]. Behnood and Mannering (2015) also found sport utility vehicles had a statistically significant effect on driver injury severities [8].

Single-vehicle accidents are defined as “those not involving any other road users other than the driver of one vehicle” [9]. Single- and multi-vehicle accidents share different risk factors and mechanisms leading to accident occurrence, and so they need to be separately described and analyzed [4,6]. Moreover, single-vehicle crashes are often implied to be due to errors only of the driver [7]. Hence, drivers’ characteristics appear to be more crucial in single-vehicle crashes than in multi-vehicle crashes. 

Single-vehicle crashes are also found to have more fatalities than multi-vehicle crashes, as was evidenced by the fact that single-vehicle crashes accounted for 28.9% of all crashes, but 58.1% of all fatal crashes in the U.S. in 2015 [10]. However, no study has been conducted in Taiwan to analyze the rates of injury and fatality according to vehicle type, neither gender- nor age-specific in single vehicle crashes, mainly due to previous unavailability of population-based data. Hence, this study aimed to use nationwide population-based administrative data to describe demographic characteristics and rates of injury and fatality among victims involved in single-vehicle crashes in Taiwan.

## 2. Materials and Methods

### 2.1. Source of Data and Study Design

This study was a descriptive study that depicted demographic characteristics of driver victims involved in single-vehicle crashes in Taiwan. The study compared rates of injury and fatality between driver victims with different vehicle types. Data analyzed were retrieved from the Police-reported Traffic Accident Registry (PTAR) from January 1 to December 31, 2016, provided by the National Policy Agency, Ministry of the Interior of Taiwan. In Taiwan, the vehicle accident investigators are qualified police who must follow the Act of “Regulation Governing Road Traffic Accidents” in handling road traffic accidents [11].

The PTAR comprises profiles for “accidents” and “victims,” and it is recorded by vehicle accident investigators from local police departments. The accident profile contains general information on individual vehicle accidents, such as date of accident, a unique accident identification number, and causes of traffic accident. The victim profile records the role of driver, passenger, or pedestrian, date of birth, gender, type of vehicle (bus, truck, car, motorcycle, or bicycle), with/without injury at the time of crash, dead/alive within 30 days, and personal identification number of each victim involved in the accident. Despite one or more victims possibly being involved in a single accident, only driver victim is analyzed in this study. Accident and victim profiles can be inter-linked by using the following three variables: date of accident, accident identification number, and identification number of the vehicle accident investigator in charge of the accident.

### 2.2. Statistical Analysis

We retrieved records for all driver victims involved in single-vehicle crashes in 2016 (*n* = 40,210), which accounted for 8.0% of all driver victims (502,540) involved in vehicle crashes in Taiwan. Age on the date of accident for driver victim was categorized into five groups: 0–17, 18–29, 30–44, 45–64, and above 65 years old. We first presented overall and age- or sex-specific number and proportion of driver victims according to vehicle type. Then, we calculated overall and age- or sex-specific rates of all-cause injuries among driver victims and fatality rate for those injured. Injury and fatality rates were also stratified according to vehicle type and presented graphically by Tableau (Tableau Software, Seattle, Washington).

This study was approved by the Institutional Ethics Review Board of National Cheng Kung University Hospital (A-EX-107-009). All study subjects analyzed in this study were anonymous, and on-site analyses were performed at the Health and Welfare Data Science Centre (HWDSC) supervised by the Ministry of Health and Welfare, Taiwan.

## 3. Results

**Figure 1:**
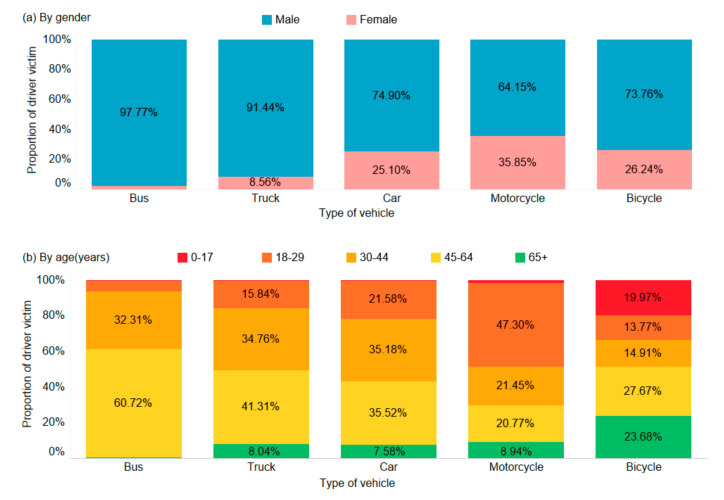
Proportion of driver victims involved in single-vehicle crashes according to vehicle type (**a**) by gender and (**b**) by age.

Table 1 shows the demographic characteristics of all driver victims, people injured and deceased involved in single-vehicle crashes according to vehicle type in 2016. Although most driver victims were males irrespective of vehicle type, the age distributions were very dissimilar among driver victims across vehicle types (Figure 1). Bus, truck, and car driver victims were mostly 30–64 years old, whereas the majority of motorcycle rider victims were younger, at 18–29 years old. Compared with driver victims of other vehicles, bicycle rider victims tended to be more equally distributed in all age groups.

**Figure 2:**
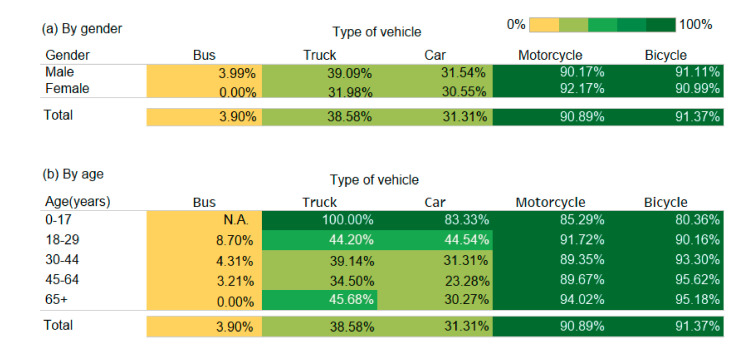
Prevalence (per 100) of injury among driver victims involved in single-vehicle crashes according to type of vehicle (**a**) by gender and (**b**) by age.

Motorcycle and bicycle rider victims had very high rates of injury (>90%). The rate of injury was low for driver victims of trucks (38.58%) and cars (31.31%) and was very low (3.90%) for bus driver victims. Similar findings were observed for gender-specific analyses. The age-specific analysis also revealed very high rates of injury for rider victims of motorcycles and bicycles. However, injury rates were very high in young (<30 years) driver victims of trucks and cars. Injury rate was also relatively high among elderly (≥65 years) truck driver victims (Figure 2). 

**Figure 3:**
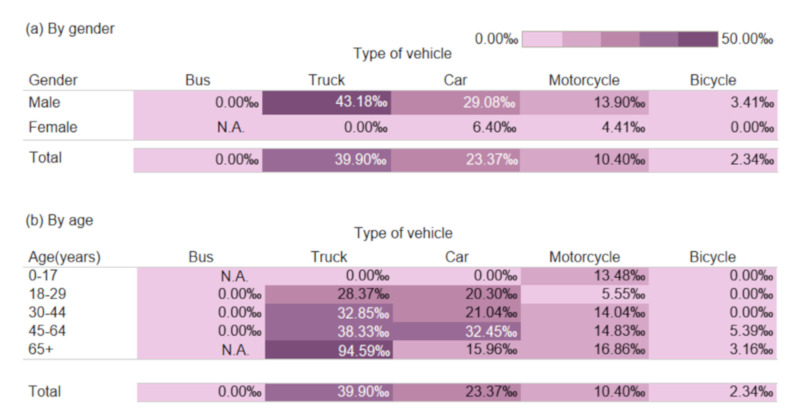
Fatality rate (per 1000) among injured driver victims involved in single-vehicle crashes according to vehicle type (**a**) by gender and (**b**) by age; N.A.: Not available.

Case-fatality rates were higher for truck (39.90‰) and car (23.37‰) driver victims but relatively low for motorcycle and bicycle riders at 10.40‰ and 2.34‰, respectively. Male victims consistently had considerably higher fatality rates than female victims for all vehicle types (Figure 3). However, the overall fatality rate was considered low for bicycle rider victims, but those with older ages (45+ years) may still be at a greater risk of death. Middle-aged and older driver victims (45+ years) also had higher fatality rates for truck and car crashes.

## 4. Discussion

### 4.1. Main Findings

Similar to previous research [12], this study noted that male and older driver victims were at a greater risk of case-fatality. We also disclosed the role of vehicle type in association with injury and case-fatality rates among driver victims involved in single-vehicle crashes. Although truck and car driver victims had higher fatality rates, older bicycle and motorcycle rider victims also suffered from a relatively high fatality rate.

### 4.2. Sex and Age Variability of Injury and Fatality by Vehicle Type

Previous studies found that male drivers were more likely to be involved in fatal and non-fatal crashes compared with female drivers [13,14]. However, Kelly-Baker and Romano (2010) reported that in the United States the prevalence of female drivers involved in fatal motor vehicle accidents is rising, and it is decreasing among male drivers [15]. Our study showed that the injury rate was consistently higher in male drivers than in female drivers for crashes of trucks, cars, and bicycles. By contrast, female motorcycle driver victims had slightly higher injury rates than male motorcyclist victims. A recent Taiwanese study also revealed that, compared with males, female motorcyclists were slightly more subject to non-fatal injury needing hospitalization (11.3% vs. 10.3%) [16]. Motorcyclists usually require physical power to safely manage motorcycles. Women are usually less powerful than men, possibly contributing to a higher risk of injury for female motorcyclists at the time of crashes. 

Unlike injury rates that showed only a small gender difference, our study found apparently higher fatality rates in male driver victims than in females in most vehicle types, with a notably large difference in truck crashes. Male driver victims tended to present characteristics that include competition, irritability, a desire for driving fast, and an attraction to stimulation, which are likely to be associated with higher risk and severity of traffic accidents [17]. Öström and Eriksson (1993) also noted that male drivers from single-vehicle crashes more often suffered from alcohol abuse, did not wear a seatbelt, and lacked driving licenses [18]. Moreover, Cordellieri et al. (2016) also reported that both men and women were able to understand and detect risk of traffic accidents, but women showed more concern about the risk than men did [19].

Our study findings demonstrated that younger motorcyclist victims were at a higher risk of injury from single-vehicle crashes possibly due to speedy driving by younger motorcyclists, which resulted in a high proportion of speed-related incidents, a high incidence of overturns and fixed object collisions, and an increased likelihood of crashing on single-carriageway roads [20]. These findings were also consistent with the literature, that younger driver crashes frequently resulted from risk-taking behaviors and inexperience in controlling vehicles [21].

Elderly truck driver victims (above 65 years old) and middle-aged bicycle rider victims (above 45 years old) were also driver victim groups with higher injury/fatality rates. Increases in drivers’ age result in gradual declines in physiological responses. Furthermore, drivers in middle to old age tend to suffer from impaired vision, hearing, and capability in responding to the outside world. Severe consequences after vehicle crashes occurred with elderly drivers, which is likely attributable to their excess fragility [18]. Our data showed that elderly truck driver victims had a very high rate of fatality, which could be due to excess fragility among elderly truck drivers. Bener (2006) also illustrated that head injury was more common in those who had crashes in four-wheel-drive vehicles (45.6%) than those who had crashes in small cars (37.3%) [22]. Bener (2006) also reported that driving four-wheel drive vehicles at night or with other poor road conditions such as in deserts or sand dunes may cause even more severe and critical injuries, usually leading to sudden deaths and permanent disability [20]. Whether these time and road conditions related factors could have also explained a higher fatality rate among truck and car driver victims needs further investigation.

Contributions of this study also include highlighting the higher rates of injury and death concerning older people involved in vehicle crashes. According to national statistics [23], the percentage of the elderly population >65 years of age has increased from 7% in 1993 to 10.6% in 2010. It is estimated that this population in Taiwan will reach 20.1% of the total population in 2050 [24], indicating that Taiwan is among the fastest aging countries in the world. At present, there is no specific regulation for elderly drivers in Taiwan to ensure and improve the safety of driving. Our study suggests a need for a regulation such as scheduled regular vision and hearing tests, managing medication, and updating driver skills to make elderly people stay physically active for driving purposes. 

### 4.3. Study Strengths and Limitations

The strengths of this population-based study included the following. First, a single-vehicle crash is most likely due to loss of control or driving errors committed by the driver, and this study analyzed injury/fatality among driver victims from single-vehicle crashes, helping to determine the specific role and responsibility of drivers involved in the crashes. Second, the Taiwan PTAR provides comprehensive information related to vehicle crashes, such as vehicle type and death at scene. Those who died at the crash scene may not necessarily be transported to hospitals. Thus, the fatality rate might be underestimated for some hospital-based studies. Third, we graphically presented the injury and fatality rates according to various stratifications. The visualization of injury/fatality data in multiple groups is easy to follow, and readers can quickly and easily note from the intersection of the categories which one is the strongest or the weakest.

Despite the above strengths, this study also has limitations. The PTAR only records injuries at the crash scene but does not include injuries that occurred days after. Driver victims are likely to seek medical care for minor or delayed injuries several days after crashes. As such, our study is likely to underestimate the rates of overall injury and in turn overestimate fatality rates.

## 5. Conclusions

This is the first study using Taiwan nationwide population-based data to describe rates of injury and death in association with victims’ demographic characteristics according to vehicle type. Minimal differences were found in injury rates between male and female driver victims. Male driver victims showed consistently higher fatality rates than females did, irrespective of vehicle type. We also noted high fatality rates among truck and car driver victims. Additionally, despite a low fatality rate being noted among bicycle and motorcycle rider victims, those aged 45 years and older continued to experience a relatively high fatality rate. This is a simple descriptive study that included no information of many factors such as road conditions, speeding and restraint use, which makes specific interpretations of study findings difficult. The vehicle-type-specific analysis of injury and case-fatality among driver victims is considered useful in identifying driver victims who are at greater risk of injury and case-fatality from single-vehicle crashes. Because motorcyclists accounted for most victims involved in single-vehicle crashes in Taiwan, we suggested that more local research is needed to explore the factors leading to motorcycle crashes, especially for those drivers of middle and old ages.

## Figures and Tables

**Table 1:** Demographic characteristics of driver victims involved in single-vehicle crashes according to vehicle type in 2016.

	Vehicle Type														
	Bus			Truck			Car			Motorcycle			Bicycle		
	No. of Victim	No. of Injured	No. of Dead	No. of Victims	No. of Injured	No. of Dead	No. of Victims	No. of Injured	No. of Dead	No. of Victims	No. of Injured	No. of Dead	No. of Victims	No. of Injured	No. of Dead
Gender															
Male	351	14	0	1837	718	31	6106	1926	56	18,032	16,260	226	967	881	3
Female	8	0	0	172	55	0	2046	625	4	10,079	9290	41	344	313	0
Age (yrs)															
0–17	0	0	0	1	1	0	12	10	0	435	371	5	280	225	0
18–29	23	2	0	319	141	4	1769	788	16	13,356	12,250	68	193	174	0
30–44	116	5	0	700	274	9	2884	903	19	6057	5412	76	209	195	0
45–64	218	7	0	832	287	11	2912	678	22	5866	5260	78	388	371	2
65+	2	0	0	162	74	7	621	188	3	2523	2372	40	332	316	1
Total ^a^	359	14	0	2014	777	31	8198	2567	60	28,237	25,665	267	1402	1281	3

^a^ Difference between the total number by gender and age constituted uninformed data.
